# Antimicrobial, Antioxidant, Antitumor, and Anti-Inflammatory Properties of *Gleichenella pectinata,* a Bioprospecting of Medicinal Ferns

**DOI:** 10.3390/antiox14111354

**Published:** 2025-11-12

**Authors:** Elena Coyago-Cruz, Rebeca Gonzalez-Pastor, Gabriela Méndez, Mateo Moya-Coyago, Juan A. Puente-Pineda, Johana Zúñiga-Miranda, Marco Cerna, Jorge Heredia-Moya

**Affiliations:** 1 Carrera de Ingeniería en Biotecnología, Universidad Politécnica Salesiana, Sede Quito, Campus El Girón, Av. 12 de Octubre N2422 y Wilson, Quito 170143, Ecuador; 2 Centro de Investigación Biomédica (CENBIO), Facultad de Ciencias de la Salud Eugenio Espejo, Universidad UTE, Quito 170527, Ecuador; 3 Carrera de Ingeniería Mecatrónica, Universidad Politécnica Salesiana, Sede Quito, Campus Sur, Av. Rumichaca Ñan s/n, Quito 170146, Ecuador

**Keywords:** bioactive compounds, functional food, cell lines, ABTS, DPPH, star fern

## Abstract

*Gleichenella pectinata*, known as ‘Star fern’, is a species traditionally used by Amazonian indigenous communities to treat various diseases. The objective of this study was to evaluate the bioactive compounds and antioxidant, antimicrobial, antitumor, and anti-inflammatory activities of *G. pectinata* leaves. The study included the determination of physicochemical parameters (pH, soluble solids, titratable acidity, moisture, and ash), phytochemical screening, mineral analysis by atomic absorption and quantification of bioactive compounds (vitamin C, organic acids, carotenoids, chlorophylls, and phenols) by liquid chromatography (RRLC). Antioxidant (ABTS and DPPH), antimicrobial (ATCC bacteria and fungi, and multi-resistant strains), antitumor and anti-inflammatory activities were evaluated. The results showed the presence of acetogenins, high concentrations of malic acid (56,559.7 mg/100 g DW), β-carotene (266.6 mg/100 g DW), chlorophyll b (684.7 mg/100 g DW), ferulic acid (3163.5 mg/100 g DW) and quercetin glucoside (945.9 mg/100 g DW). The freeze-dried ethanolic extracts showed greater efficacy against *Pseudomonas aeruginosa* ATCC (12.0 mg/mL) and multidrug-resistant strains of *E. coli* (6.6 mg/mL) and *P. aeruginosa* (6.6 mg/mL). In addition, the extract exhibited moderate antiproliferative activity (IC_50_: 0.98–1.98 mg/mL) in hepatocellular and cervical carcinoma cell lines. In conclusion, this study provides the first evidence of the antitumor and bioactive potential of *G. pectinata*, supporting its value as a natural source of functional compounds with potential pharmacological applications.

## 1. Introduction

*Gleichenella pectinata* (Willd.) Ching (‘Star fern’) is a species of fern belonging to the *Polypodiopsida* class, *Gleicheniales* order, *Gleicheniaceae* family and *Gleichenella* genus. It is a monotypic taxon. It has various botanical synonyms, including *Gleichenia brasiliana*, *Gleichenia hermannii, Mertensia hookeri, Mertensia emarginata* and *Dicranopteris pectinata*. Members of the order *Gleicheniales* are mainly found in tropical regions and comprise between 130 and 150 species, which are grouped into three to five genera [1,2,3].

*G. pectinata* thrives in nutrient-poor soils, particularly in tropical regions, where it forms extensive colonies along roads and in other disturbed areas. Its ability to colonise new spaces efficiently is associated with the presence of creeping rhizomes and the indeterminate growth of its fronds [4].

From a phytochemical perspective, research on *G. pectinata* is limited; however, preliminary analyses have identified phenolic compounds, particularly glycosylated flavonols such as kaempferol-3-rhamnosylglucose, quercetin-3-glucose and kaempferol-3-glucose [5]. These compounds belong to the flavonoid group, which is widely recognised for its antioxidant activity and pharmacological potential [6].

Interest in this species increases when you consider that other members of the *Gleicheniaceae* family, such as *G. linearis* and *G. truncata*, have a diverse phytochemical profile and are used in traditional medicine. For example, *G. linearis* contains alkaloids, phenols, flavonoids, tannins, triterpenoids and steroids—compounds commonly associated with therapeutic effects [7]. In traditional medicine, *G. linearis* is used in powder form, as an aqueous extract, or as a hot juice to treat fertility problems, sore throats, coughs, asthma, bacterial infections, intestinal parasites, wounds, ulcers, and fever [8]. Furthermore, *G. truncata* has demonstrated anti-inflammatory and antimalarial properties by inhibiting glycogen synthase kinase-3, which is a key regulator of inflammatory responses [9].

Beyond these species, ferns are used in traditional medicine to treat ailments such as fever, wounds, infections, pain, rheumatism, respiratory diseases, and gastrointestinal problems, in preparations including infusions, decoctions, lotions, and poultices. From a scientific perspective, the flavonoid, polyphenol, polyunsaturated fatty acid, carotenoid, terpenoid and steroid content of some ferns has been characterised, and these compounds are associated with antioxidant, anti-inflammatory and antimicrobial activities [8].

Conversely, bioactive plant compounds are secondary metabolites that perform adaptive functions and have beneficial effects on human health. These include phenols, terpenoids, thiols and dietary fibres [10,11]. They act by regulating cellular redox balance, modulating inflammatory pathways, and interfering with gene expression through epigenetic mechanisms. Such properties have been linked to the prevention of chronic diseases such as cancer, cardiovascular disease and metabolic disorders [12].

Several flavonoids, such as quercetin and epigallocatechin gallate, are among the compounds most studied for their biological activity. These compounds have demonstrated the ability to inhibit bacterial toxins, such as haemolysins, including the thermolabile haemolysin of *Vibrio parahaemolyticus*. This reduces their haemolytic activity [13]. Similarly, various phenolic acids, such as gallic acid and cinnamic acid, have been shown to possess antibacterial properties [14]. In particular, quercetin is effective against *Staphylococcus aureus* [15], while rutin and catechin have been effective against *Escherichia coli* and *Pseudomonas aeruginosa* [16]. Kaempferol, myricetin and quercetin have also been studied for their potential anti-tumor properties, particularly in breast and gynaecological cancers [17].

In this context, given the reported presence of flavonoids in the genus *Gleichenella* and the observed therapeutic potential of related species, this study aimed to evaluate the bioactive compounds and antioxidant, antimicrobial, antitumor and anti-inflammatory activities of *Gleichenella pectinata* leaves. These fern species have received little scientific attention despite their promising potential, so the results will serve as a starting point for future research.

## 2. Materials and Methods

### 2.1. Plant Material and Physico-Chemical Analysis

**Figure 1:**
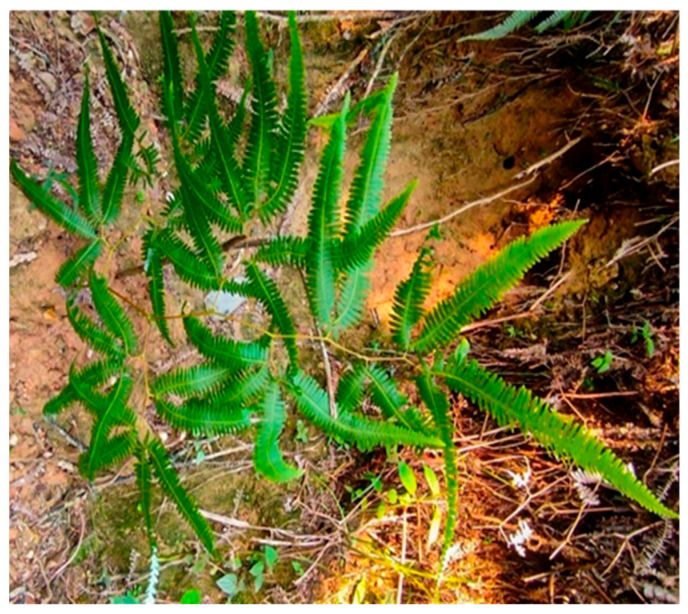
Photograph of *G. pectinata* leaves (‘Star fern’).

The study collected ‘*Star fern*’ (*Gleichenella pectinata*) leaves in the Amazon region of the Ecuador province of Pastaza (1°44′21″ S, 77°29′1″ W) in March (Figure 1). For botanical species identification, the plant was analysed at the herbarium at the Salesian Polytechnic University in Quito, Ecuador. Fresh plant material was stored between layers of paper and cardboard to preserve structural integrity. The leaves were collected randomly from different plants in the same area. The sample was divided into two parts; thus, the first part, when fresh, was used for physical and chemical characterisation, while the second part was kept in sterile conical centrifuge tubes and maintained at −80 °C until lyophilisation. Freezy-drying was carried out using a Christ Alpha 1–4 LDplus system (GmbH, Osterode am Harz, Germany). Once dried, the samples were ground into a homogeneous fine freeze-dried ‘Star fern’, transferred into amber glass containers under a nitrogen atmosphere, and stored at −20 °C for subsequent analysis.

Physicochemical characterisation involved the determination of various parameters, including pH, moisture and ash content, soluble solids content, and titratable acidity. The pH was measured using SevenMultiS47 (Mettler Toledo, Columbus, OH, USA). Soluble solids were assessed with a Hitech RHB-32 refractometer (G-Won Hitech Co., Ltd., Seoul, Republic of Korea). Titratable acidity was determined by acid-base titration with 0.1 M NaOH. Moisture content was calculated by oven drying at 100 °C using a Memmert Be 20 device (Memmert GmbH + Co.KG, Barcelona, Spain) until reaching constant weight. In contrast, ash content was obtained after calcination in a muffle (Thermo Fisher Scientific, Waltham, MA, USA) at 550 °C [18].

### 2.2. Mineral Quantification

For the extraction of macro (potassium, calcium, sodium, and magnesium) and micro (iron) mineral content, 40 mg of freeze-dried ‘Star fern’ was transferred into vessels of a Speedwave Xpert microwave digestion system (Berghof products + Instruments GmbH, Eningen unter Achalm, Germany) [18]. Subsequently, 5 mL of concentrated nitric acid (65%) was added to each vessel. The mixtures were allowed to stand for 10 min to ensure initial acid-sample interaction before sealing the vessel. The mixtures were allowed to stand for 10 min to ensure initial acid-sample interaction before sealing the vessels. Microwave-assisted digestion was carried out using a three-step temperature and pressure program. First, a linear ramp to 140 °C under 30 bars at 70% power for 5 min; followed by heating to 200 °C under 35 bars at 80% power for 15 min; and finally, cooling to 50 °C under 25 bars with no power input over 10 min. Upon completion, the digestion vessels were cooled to ambient temperature for 20 min. Each digested sample was diluted to a final volume of 25 mL with ultrapure water (Mili-Q) and stored in amber glass bottles until instrument analysis.

Mineral analysis was conducted using an atomic absorption spectrophotometer Varian SpectrAA-55 instrument (Agilent Technologies, Santa Clara, CA, USA) [18]. Calcium was measured at 422.7 nm with a 0.5 nm slit width using an acetylene-nitrous oxide flame. At the same time, iron, sodium, potassium, and magnesium were analysed with an air-acetylene flame at wavelengths of 372.0, 589.6, 404.4, and 202.6 nm, respectively, with slit widths of 0.20, 0.5, 0.5, and 1.0 nm. Calibration was performed using dilutions of a 1000 ppm standard, with working ranges of 0–5 ppm for Ca, 0–20 ppm for Fe, 0–200 ppm for K, 0–10 ppm for Mg, and 0–8 ppm for Na. The minerals were extracted in triplicate and read in duplicate. Results are expressed as mean ± standard deviation. Results were expressed as milligrams per 100 g of dry weight of ‘Star fern’ (mg/100 g DW).

### 2.3. Screening Phytochemical

The extract was prepared by weighing 20 mg of freeze-dried ‘Star fern’ and initially mixing it with one mL of deionised water. The mixture was homogenised in a VM-300 vortex device (Interbiolab Inc., Orlando, FL, USA), followed by ultrasonication in an FS60 ultrasonic bath (Fisher Scientific Inc., Waltham, MA, USA) for 3 min at room temperature (approximately 25 °C) and at a frequency of 40 kHz. The supernatant and residue were separated in a 5430 microcentrifuge (Eppendorf AG, Hamburg, Germany) at 14,000 rpm, 4 °C, and 5 min. The supernatant was saved, and the residue was re-extracted twice more using 500 µL of water.

The final supernatant was used to detect the presence or absence of secondary metabolites according to the methodology described by León-Fernández et al. [19]. Thus, terpenoids were analysed by reaction with chloroform and sulphuric acid; phenols and tannins with 10% ferric chloride; alkaloids with 2 N hydrochloric acid and Mayer’s reagent; flavonoids with a 10% 1 M ammonia solution and concentrated sulphuric acid; and anthraquinones with benzene and 10% ammonia. The presence of foam was used as an indicator of saponins, and acetogenins were confirmed with 3,5-dinitrobenzoic acid and potassium hydroxide. All extractions were performed in triplicate, and readings were taken in duplicate. The results were recorded qualitatively as ‘+’ for presence and ‘-’ for absence of the compound.

### 2.4. Bioactive Compounds

#### 2.4.1. Vitamin C Identification

*L*-ascorbic acid (1 mg/mL) was used as a reference standard for vitamin C quantification [18]. A sample of 20 mg of freeze-dried ‘Star fern’ was dissolved in 1.2 mL of 3% metaphosphoric acid and 200 µL of 0.2% *DL*-homocysteine. The mixture was vortexed and sonicated for one minute. Following homogenization, 600 mL of ultrapure water was added, and the solid was separated for centrifugation (14,000 rpm, 4 °C, 5 min). The supernatant was filtered before analysis with a 0.45 µm PVDF filter. Chromatographic separation was carried out using a rapid resolution liquid chromatography (RRLC) 1200 system (Agilent Technologies, Mississauga, ON, Canada) equipped with a DAD-UV-VIS detector at 244 nm, and a Zorbax Eclipse XDB 80 AC C18 column (1.8 μm, 4.6 mm × 50 mm) (Agilent Scientific Instruments, Santa Clara, CA, USA). The mobile phase consisted of a solution prepared in a 90:10 (*v*/*v*) ratio of two solutions, the first being an aqueous solution of 1.5% (*w*/*v*) KH2PO4 and the second being a methanolic solution of 1.8% (*w*/*v*) n-cetyl, n, n, n-trimethylammonium bromide. The mobile phase was pumped at a flow rate of 1 mL/min. The chromatograms were recovered and analysed using Lab ChemStation software version 2.15.26 (Agilent Technologies, Santa Clara, CA, USA) comparing the retention times of the standard, the spectrum of *L*-ascorbic acid and an internal standard. The extraction was performed in triplicate, and the injection was performed in duplicate for each sample. Results are expressed as mean ± standard deviation. Results were reported as milligrams of vitamin C per 100 g of dry weight of ‘Star fern’ (mg/100 g DW).

#### 2.4.2. Organic Acid Identification

References standards at a concentration of 100 mg/mL of tartaric, malic, and citric acids were employed for the identification and quantification of individual organic acids [18]. For the extract, 20 mg of the freeze-dried ‘Star fern’ was suspended in 1.5 mL of 0.02 M sulfuric acid supplemented with 0.05% metaphosphoric acid and 0.02% *DL*-homocysteine. The mixture was vortexed and sonicated for three minutes. The supernatant recovered for centrifugation (14,000 rpm, 4 °C, 5 min) was filtered before analysis with a 0.45 µm PVDF filter and analysed with an RRLC 1200, DAD-UV-VIS detector at 210 nm and a YMC-Triart C18 column (3 µm, 4.6 mm × 150 mm) (YMC Europe GmbH, Dinslaken, Germany). The mobile phase consisted of a 0.027% sulfuric acid solution and was pumped at a flow rate of 1 mL/min. The chromatograms were recovered and analysed using Lab ChemStation software version 2.15.26, which compared the retention times of the standard, the spectrum of organic acids, and an internal standard. The extraction was performed in triplicate, and the injection was performed in duplicate for each sample. Results are expressed as mean ± standard deviation. Results were reported as milligrams of individual organic acid per 100 g of dry weight of ‘Star fern’ (mg/100 g DW).

#### 2.4.3. Carotenoids Identification

Reference standards at a concentration of 1 mg/mL of astaxanthin, α-carotene, β-carotene, β-crytoxanthin, lutein, lycopene, trans-β-apo-8-carotenal, and zeaxanthin were employed for the identification and quantification of individual carotenoids [18]. For extraction, 20 mg of freeze-dried ‘Star fern’ was combined with 250 µL of acetone, 500 µL of dichloromethane, and 250 µL of methanol. The mixture was vortexed and sonicated for two minutes. The coloured liquid was recovered by centrifugation (14,000 rpm, 4 °C, 5 min), and the solid was re-extracted with 500 µL of the mixture until the pigment was completely recovered from the sample. The resulting-coloured liquid was concentrated to dryness under reduced pressure using a rotary evaporator Buchi TM R-100 (Fisher Scientific, Hampton, NH, USA) at a temperature below 30 °C. The dry pigment was reconstituted in 40 µL of ethyl acetate, which was centrifuged (14,000 rpm, 4 °C, 5 min), and 20 µL was placed in a vial equipped with an insert for the respective chromatographic analysis in an RRLC 1200 equipped with a DAD-UV-VIS detector and a YMC C30 column (3 µm, 4.6 × 150 mm) (YMC Europe GmbH, Dinslaken, Germany). A ternary mobile phase composed of methanol (solvent A), methyl tert-butyl ether (solvent B), and water (solvent C) was pumped at 1 mL/min employed under a linear gradient program, thus 95% A + 5% B + 0% C (0–5 min), 89% A + 11% B + 10% C (10 min), 75% A + 25% B + 0% C (16 min), 40% A + 60% B + 0% C (20 min), 15% A + 85% B + 0% C (22.5 min), and 90% A + 5% B + 5% C (25–28 min). The chromatograms were recovered at 350 or 450 nm and analysed using Lab ChemStation software version 2.15.26, comparing the retention times of the standard, the spectrum of carotenoids and an internal standard. The extraction was performed in triplicate, and the injection was performed in duplicate for each sample. Results are expressed as mean ± standard deviation. Results were reported as milligrams of individual carotenoids per 100 g of dry weight of ‘Star fern’ (mg/100 g DW).

#### 2.4.4. Chlorophylls and Their Derivatives

Reference standards at a concentration of 1 mg/mL of chlorophyll a, pheophytin a, and pheophytin b were employed for the identification and quantification of individual compounds [18]. The extraction and quantification of chlorophyll pigments and their degradation derivatives were performed following the methodology previously detailed (Section 2.4.3). The extraction was performed in triplicate, and the injection was performed in duplicate for each sample. Results are expressed as mean + standard deviation. Results were reported as milligrams of individual chlorophylls and their derivatives per 100 g of dry weight of ‘Star fern’ (mg/100 g DW).

#### 2.4.5. Phenolic Compound Identification

Reference standard at a concentration of 1 mg/mL of caffeic acid, vanillic acid, chlorogenic acid, syringic acid, chrysin, shikimic acid, *p*-, *m*-, and *o*-coumaric acids, rutin, 2,5-dihydroxybenzoic acid, quercetin, ferulic acid, naringin, gallic acid, luteolin, *p*-hydroxybenzoic acid, kaempferol, 2,5-dihydroxybenzoic acid, and 3-hydroxybenzoic acid were employed for the identification and quantification of individual phenolics [18].

For extraction, 20 mg of freeze-dried ‘Star fern’ was combined with 1000 µL of 80% methanol acidified with 0.1% HCl. The mixture was vortexed and sonicated for three minutes. The liquid was recovered by centrifugation (14,000 rpm, 4 °C, 5 min), and the solid was re-extracted with 500 µL of methanol acidified twice more. The methanolic extract was filtered with a 0.45 µm PVDF filter and analysed with an RRLC 1200, DAD-UV-VIS detector between 220 nm and 500 nm, and a Zorbax Eclipse Plus C18 column (4.6 × 150 mm, 5 µm) (Agilent Scientific Instruments, Santa Clara, CA, USA). A binary mobile phase was pumped at 1 mL/min, consisting of an aqueous solution with 0.01% formic acid, and solvent B was acetonitrile. The elution gradient was programmed with initial conditions at 100% A, transitioning to 95% A and 5% B at 5 min, followed by a gradual shift to 50% A and 50% B at 20 min, and maintained until 30 min. After this, the column was washed for two minutes and re-equilibrated. The chromatograms were recovered at 280 nm, 320 nm, or 370 nm, depending on each compound, and analysed using Lab ChemStation software, comparing the retention times of the standard, the spectrum of phenolics and an internal standard. The extraction was performed in triplicate, and the injection was performed in duplicate for each sample. Results are expressed as mean ± standard deviation. Results were reported as milligrams of individual phenolics per 100 g of dry weight of ‘Star fern’ (mg/100 g DW).

### 2.5. Antimicrobial Activity

Phenolic-rich extract was prepared by suspending 2.5 g of freeze-dried ‘Star fern’ (*Gleichenella pectinate*) in 25 mL of 50% ethanol, followed by homogenization and sonication for 6 min. The liquid was recovered by centrifugation (7500 rpm, 5 min, 4 °C), and the solid was re-extracted with 25 mL of ethanolic solution twice more. The ethanolic extract was filtered with filter paper, and ethanol was evaporated under reduced pressure at temperatures not exceeding 40 °C. The concentrated extracts were frozen and lyophilised. The freeze-dried ethanolic extract was stored frozen until analysis. This extraction method was previously validated by Coyago et al., maximising the concentration of phenolic compounds, which are the metabolites that mainly generate biological activities [20].

#### 2.5.1. Antibacterial Activity

The antibacterial activity was evaluated using the agar well diffusion method and the broth microdilution technique to determine the minimum inhibitory concentration (MIC), following protocols outlined by the Clinical and Laboratory Standards Institute (CLSI) [21,22,23]. Freeze-dried extracts were tested against *Streptococcus aureus* ATCC 6538P, *Streptococcus mutans* ATCC 25175, *Escherichia coli* ATCC 8739, and *Pseudomonas aeruginosa* ATCC 9027, obtained from the American Type Culture Collection.

Before testing, 300 mg of freeze-dried ethanolic extract were reconstituted in 1 mL of sterile distilled water. Bacterial cultures were maintained on solidified Mueller–Hinton agar (MHA). For the agar well diffusion assay, microbial inoculation was adjusted to approximately 1.5 × 10^8^ CFU/mL and spread uniformly onto agar plates. Wells with 5 mm diameters were created on the agar surface, and 80 µL of each extract was added to the wells. Streptomycin sulphate salt was used as a positive control for bacterial strains. Each test was performed in triplicate. Plates were incubated at 37 °C for 24 h, after which inhibition zones were measured in millimetres. Results are expressed as mean ± standard deviation.

For the microdilution technique, 100 µL of each freeze-dried ethanolic extract in solution was combined with 100 µL of Brain Heart Infusion (BHI) medium in a sterile 96-well microplate, and serial twofold dilutions were prepared directly within the wells. Subsequently, 20 µL of bacterial suspension, standardised to 5 × 10^5^ CFU/mL, were added to each well, yielding a final reaction volume of 220 µL. Streptomycin sulphate salt was included as the positive control, while wells containing the bacterial suspension and sterile water served as the negative control. The microplates were incubated at 37 °C for 24 h. To visualise bacterial growth, 20 µL of 2,3,5-triphenyltetrazolium chloride (TTC) was added and incubated for an additional two hours at 37 °C. Reduction of TTC to red formazan by metabolically active bacteria enabled direct visual determination of growth inhibition. The minimum inhibitory concentration (MIC) corresponded to the lowest extract concentration showing no visible red colouration. All experiments were performed at least in triplicate.

#### 2.5.2. Antibacterial Activity Against Multidrug-Resistant Bacteria

The antibacterial potential of the freeze-dried ethanolic extract of ‘Star fern’ was investigated against seven multidrug-resistant bacterial strains, such as *Klebsiella pneumoniae*, *Escherichia coli*, *Salmonella enterica* serovar Kentucky, *Enterococcus faecalis*, *Staphylococcus epidermidis*, *Enterococcus faecium*, and *Pseudomonas aeruginosa*. These isolates were obtained from the National Health Institute of Ecuador (INSPI) and are part of the institution’s External Quality Evaluation Program.

Bacterial inocula were cultured in brain heart infusion (BHI) broth and standardised to 5 × 10^5^ CFU/mL. The freeze-dried ethanolic extract was prepared in DMSO at 267 mg/mL, while nourseothricin (100 µg/mL) served as the reference antimicrobial control. BHI medium alone and BHI containing the extract at stock concentration were used as blank controls. The antibacterial assay was performed using the microdilution technique, following CLSI guidelines [23] with slight modifications. Specifically, 5 µL of the extract stock solution was added to 195 µL of bacterial suspension (5 × 10^5^ CFU/mL), reaching a total volume of 200 µL per well. The microplates were incubated at 37 °C for 20 h with continuous agitation at 300 rpm (double orbital setting). Bacterial growth inhibition was determined by measuring absorbance at 600 nm. All experiments were performed at least in triplicate.

#### 2.5.3. Antifungal Activity

The antifungal activity was evaluated using the agar well diffusion method and the broth microdilution technique to determine the minimum inhibitory concentration (MIC), following protocols outlined by the Clinical and Laboratory Standards Institute (CLSI) {Formatting Citation}. Freeze-dried ethanolic extracts were tested against *Candida albicans* ATCC 10231 and *Candida tropicalis* ATCC 13803, obtained from the American Type Culture Collection.

Before testing, 300 mg of freeze-dried ethanolic extract was reconstituted in 1 mL of sterile distilled water. Yeast strains were preserved on Sabouraud dextrose agar (SDA). For the agar well diffusion assay, microbial inoculation was adjusted to approximately 5 × 10^5^ CFU/mL and spread uniformly onto agar plates. Wells with 5 mm diameters were created on the agar surface, and 80 µL of each extract was added to the well. Fluconazole was used as a positive control for fungal strains. Each test was performed in triplicate. Plates were incubated at 35 °C for 48 h, after which inhibition zones were measured in millimetres. Results are expressed as mean ± standard deviation.

For the microdilution technique, 100 µL of each freeze-dried ethanolic extract solution was combined with 100 µL of Yeast Peptone Dextrose Broth (YPDB) medium in a sterile 96-well microplate, and serial twofold dilutions were prepared directly within the wells. Subsequently, 20 µL of fungal suspension, standardised to 1 × 10^6^ CFU/mL, was added to each well, yielding a final reaction volume of 220 µL. Fluconazole was included as the positive control, while wells containing the fungal suspension and sterile water served as the negative control. The microplates were incubated at 37 °C for 72 h. To visualise fungal growth, 20 µL of 2,3,5-triphenyltetrazolium chloride (TTC) was added and incubated for an additional two hours at 37 °C. Reduction of TTC to red formazan by metabolically active fungi enabled direct visual determination of growth inhibition. The minimum inhibitory concentration (MIC) corresponded to the lowest extract concentration, showing no visible red colouration. All experiments were performed at least in triplicate.

### 2.6. Antioxidant Activity

Reference standards at a concentration of 10 mM of Trolox were employed for quantifying antioxidant activity using the DPPH and ABTS methods. For extraction, 20 mg of freeze-dried ‘Star fern’ was combined with 2 mL of methanol. The mixture was vortexed and sonicated for three minutes. The supernatant recovered for centrifugation (14,000 rpm, 4 °C, 5 min) was filtered before analysis with a 0.45 µm PVDF (Polyvinylidene fluoride) filter (Sigma-Aldrich, Darmstadt, Germany) [18].

The DPPH• radical solution was freshly prepared by dissolving 10 mg of DPPH in 50 mL of methanol. For calibration curve preparation, Trolox standard solutions ranging from 0.1 to 4.0 mM were prepared in methanol. For each reaction, 20 µL of sample or standard was added to 280 µL of DPPH• solution in a microplate. This was incubated in the dark for 40 min on an orbital shaker 4310 (Fisher Scientific, Waltham, MA, USA). Absorbance readings were taken at 515 nm using a BioTek H1 spectrophotometer (Agilent Scientific Instruments, Santa Clara, CA, USA).

The ABTS•+ radical cation was generated by mixing equal volumes of 7 mM ABTS and 2.45 mM potassium persulfate solutions, followed by incubation in the dark for 16 h. Before use, the radical solution was diluted tenfold with absolute methanol until reaching an absorbance of approximately 0.7 at 734 nm. For calibration curve preparation, Trolox standard solutions ranging from 0.2 to 0.7 mM were prepared in ethanol. For each reaction, 20 µL of sample or standard was added to 280 µL of ABTS•+ radical in a microplate. This was incubated in the dark for 15 min on an orbital shaker. Absorbance readings were taken at 734 nm in a spectrophotometer.

The extraction was performed in triplicate, and the quantification in duplicate. Results are expressed as mean ± standard deviation. Results were expressed as millimoles of Trolox equivalents per 100 g of dry weight of freeze-dried ‘Star fern’ (mmol TE/100 g DW).

### 2.7. Antitumor Activity

The cell lines HeLa (human cervical carcinoma, ATCC No. CCL-2, RRID: CVCL_0030), HCT116 (human colorectal carcinoma, ATCC No. CCL-247, RRID: CVCL_0291), HepG2 (human hepatoma, ATCC No. HB-8065, RRID: CVCL_0027), and NIH3T3 (mouse embryonic fibroblasts, ATCC No. CRL-1658, RRID: CVCL_0594) were sourced from the American Type Culture Collection (ATCC, Manassas, VA, USA). Additionally, the THJ29T cell line (human thyroid carcinoma, Cat. No. T8254, RRID: CVCL_W922) was procured from Applied Biological Materials Inc. (ABM, Richmond, BC, Canada). This panel was intentionally assembled to cover distinct tissue origins and molecular backgrounds, enabling a wider appraisal of the extract’s antiproliferative effects. All cell cultures were maintained in Dulbecco’s Modified Eagle’s Medium/Nutrient Mixture F-12 Ham (DMEM/F12) (Corning, Manassas, VA, USA) supplemented with 10% fetal bovine serum (FBS) (Eurobio, Les Ulis, France) and 1% penicillin-streptomycin (Thermo Fisher Scientific, Gibco, Miami, FL, USA). Cultures were kept at 37 °C in a humidified incubator with 5% CO_2_.

To assess the impact of the freeze-dried ethanolic extract of *G. pectinata* (Section 2.5) on cell proliferation, cells were plated in 96-well plates at a density of 1 × 10^4^ cells per well. After allowing 24 h for cell attachment, the cultures were exposed to 100 µL of the extract, prepared in distilled water, using serial two-fold dilutions spanning from 5 mg/mL to 0.08 mg for 72 h. This range was selected considering the extract’s solubility limits and the maximal solvent load compatible with cell viability. The 72 h exposure was chosen to align with typical doubling times and to capture cytostatic effects commonly assessed in this assay window. Cisplatin (CDDP) was used as a positive control.

Cell viability was then evaluated using the MTT assay, following standard procedures. Briefly, 10 µL of MTT (M5655, Sigma-Aldrich, Darmstadt, Germany) solution (5 mg/mL) was added to each well, and the plates were incubated for 1 to 2 h (cell line-dependent) at 37 °C under humidified conditions. The supernatant was carefully removed, and 50 µL of DMSO was added to dissolve the formed formazan crystals. Plates were placed on an orbital shaker for 2 min to ensure complete solubilization before measuring absorbance at 570 nm using the Cytation 5 multi-mode plate reader (BioTek, Winooski, VT, USA). Wells containing culture medium only served as the negative control (defined as 100% proliferation), and percent inhibition was calculated relative to this control. Each concentration was tested in quadruplicate, and experiments were independently repeated at least four times. The half-maximal inhibitory concentration (IC_50_) was calculated from dose–response curves generated using GraphPad Prism 10.2 (GraphPad Software, Corp., San Diego, CA, USA). IC_50_ values were obtained by nonlinear regression using a four-parameter logistic model. The therapeutic index (TI) was determined by dividing the IC_50_ value of non-tumour NIH3T3 cells by the IC_50_ values of each tumour cell line, with TI values > 1 interpreted as preferential activity against tumour cells. NIH3T3 was selected as the non-tumour comparator as a fibroblast model of healthy tissue. Results are expressed as mean ± standard deviation.

### 2.8. Anti-Inflammatory Activity

RAW 264.7 murine macrophages (ATCC No. TIB-71, RRID: CVCL_0493) were grown in Dulbecco’s Modified Eagle Medium (DMEM; Corning, Manassas, VA, USA) containing 4.5 g/L glucose and *L*-glutamine, supplemented with 10% fetal bovine serum (FBS; Eurobio, Les Ulis, France) and 1% penicillin-streptomycin (Thermo Fisher Scientific, Gibco, Miami, FL, USA). To ensure adequate growth conditions, cells were maintained using a combination of 70% fresh medium and 30% conditioned medium.Cultures were incubated at 37 °C in a humidified atmosphere containing 5% CO_2_.

For the anti-inflammatory assay, RAW 264.7 cells were seeded in 24-well plates at a density of 3 × 10^5^ cells per well and allowed to adhere for 18–24 h. Once attached, the culture medium was replaced with serum-free DMEM containing the different treatments. These included DMEM alone as the control, dexamethasone (DEX; Sigma-Aldrich, St. Louis, MO, USA) at 2 µg/mL as the anti-inflammatory reference, and freeze-dried ethanolic extract of *G. pectinate* (Section 2.5) at concentrations of 0.05 mg/mL and 0.1 mg/mL. After four hours of exposure, lipopolysaccharide (LPS; InvivoGen, San Diego, CA, USA) at 1 μg/mL was added to stimulate nitric oxide (NO) production, and the cultures were further incubated for 20 h. All stock solutions were freshly prepared in DMEM.

Nitric oxide levels were determined by transferring 50 µL of supernatant from each treatment into a 96-well plate, together with a NO standard curve prepared with Promega reagent (G296A) in the range of 0.78–100 µM. An equal volume of Griess reagent (Sigma-Aldrich, Darmstadt, Germany, G4410; 50 mg/mL) was then added to each well, giving a final reaction volume of 100 µL. After incubation for 10 min at room temperature in the dark, absorbance was measured at 540 nm using a Cytation 5 plate reader (BioTek, Vermont, VT, USA). The absorbance corresponding to DMEM alone was subtracted, and NO concentrations were calculated from the standard curve. All assays were performed in triplicate.

Cell viability was evaluated in parallel by fixing the cells in the 24-well plates with 4% paraformaldehyde for 20 min at room temperature, followed by staining with 0.5% (*w*/*v*) crystal violet for 30 min. Plates were then carefully washed with water to remove excess dye and air-dried. The stained plates were scanned, and the absorbance was measured at 570 nm using a Cytation 5 multimode detection system (BioTek, Vermont, VT, USA). Untreated cells were considered the reference for 100% viability.

## 3. Results and Discussion

### 3.1. Physico-Chemical Analysis

Table 1 shows the results of the chemical analysis of ‘Star fern’ (*Gleichenella pectinata*), including pH level, total soluble solids content, titratable acidity, moisture content, and ash content. Additionally, the mineral profile is presented, including macro (potassium, calcium, sodium, and magnesium) and micro (iron) mineral content.

The chemical results obtained for *G. pectinata* leaves reveal a characteristic profile that reflects their ecological adaptability. The slightly acidic pH value of 4.6 is consistent with that reported for other plant species growing in acidic tropical soils [24]. This acidity may have ecological and functional implications as it influences the availability of essential nutrients, such as phosphorus, calcium, and magnesium, in plants, and contributes to tolerance of adverse soil conditions [25].

The low soluble solids content (1.0 °Brix) suggests a low concentration of simple sugars, indicating a plant metabolism that is focused on synthesising secondary metabolites such as phenolic compounds and flavonoids. These compounds perform defensive functions and are associated with therapeutic properties [26,27].

Titratable acidity (2.4%) was higher than in fruits such as *Inga edulis* (0.1%), approaching values reported in more acidic species such as *Solanum sessiliflorum* (3.8%). This acidity contributes to the sensory profile and natural antimicrobial activity by limiting the growth of pathogenic microorganisms. From a physiological point of view, acidity in species such as ferns may be related to mechanisms of adaptation to abiotic stress and the solubilisation of specific metal ions [8,28].

In terms of mineral content, the analysis reveals high concentrations of potassium (877.5 mg/100 g DW), the most abundant macromineral. This is a significant result for both plant physiology, where potassium participates in osmotic regulation, photosynthesis, and stomatal closure, and human nutrition, as it is an essential mineral for neuromuscular function, blood pressure, and water balance [29]. Calcium (245.4 mg/100 g DW) was also present in significant amounts. This macronutrient is involved in maintaining the structural stability of cell walls, intracellular signalling and the response to pathogens in plants [30]. One of the most notable findings was the high concentration of iron (95.2 mg/100 g DW), which is much higher than that found in many other edible plants. This concentration could have therapeutic value in populations with a high prevalence of iron deficiency anaemia [31].

Similarly, the magnesium content (47.4 mg/100 g DW) was substantial. This mineral is directly linked to the structure of chlorophyll. It plays a role in over 300 enzymatic reactions in humans, including those involved in energy metabolism, muscle contraction and cardiovascular health [32]. Finally, *G. pectinata* exhibited a very low sodium content of 0.2 mg/100 g DW. This is favourable from both a nutritional and a functional perspective. Excess sodium intake has been linked to cardiovascular disease, and low sodium content in plants may be related to salt stress tolerance mechanisms [33].

Table 2 presents a phytochemical screening of ‘Star fern’, showing the qualitative analysis of acetogenins, alkaloids, anthraquinones, flavonoids, phenols, saponins, steroids, tannins and terpenoids. The results showed the presence of acetogenins, flavonoids, and phenols. Thus, acetogenins are secondary metabolites derived from fatty acids, which have anti-tumour activity and are found in large quantities in the Annona family [34].

### 3.2. Bioactive Compounds

Bioactive compounds are secondary plant metabolites that have beneficial effects on human health, in addition to their physiological functions in the plant [11]. Table 3 shows the profile of bioactive compounds of ‘Star fern’, including vitamin C, and the profile of organic acids, presenting the concentrations of citric, malic, tartaric acids, and total organic acid as the sum of individual compounds, as well as the profiles of carotenoids and total carotenoids (sum of individual carotenoids). The profiles of chlorophylls and their derivatives are total (sum of individual compounds), and finally, the phenolic profile and total phenolics (sum of individual phenolics).

Among the compounds identified, vitamin C was found at a concentration of 16.6 mg/100 g DW, which is comparable to that found in tropical fruits such as *Inga insignis* (16.9 mg/100 g DW) [35]. While this value is lower than the recommended daily intake of 200 mg for effective cardioprotective action [36], it remains significant as an antioxidant cofactor that synergises with other compounds in the plant matrix.

In terms of organic acids, *G. pectinata* leaves showed a predominance of malic acid (56,559.7 mg/100 g DW), followed by citric acid (162.1 mg/100 g DW), with a low concentration of tartaric acid (14.4 mg/100 g DW) also present. The total content (56.7 mg/100 mg DW) falls within the range reported for tropical fruits (1.5–80.5 mg/100 mg DW) [35], highlighting the leaf’s richness in functional organic compounds. In addition to being a key intermediate in the Krebs cycle, malic acid has demonstrated beneficial properties in improving energy metabolism, skin health, and certain medical conditions such as fibromyalgia and chronic fatigue [37]. These results are consistent with those reported for the titratable acidity of the leaf, which also showed high concentrations.

Chromatographic analysis revealed various carotenoids to be present, most notably β-carotene (266.6 mg/100 g DW), followed by lutein (112.9 mg/100 g DW) and violaxanthin (15.4 mg/100 g DW). The total carotenoid content (336.2 mg/100 g DW) was comparable to that observed in *Sicana odorifera*, a pigment-rich tropical squash (321.6 mg/100 g DW) [35]. β-Carotene, a precursor of vitamin A, is well known for its positive effects on eye health and immune function, as well as for its role as a lipophilic antioxidant. It is capable of neutralising free radicals and reducing the risk of chronic diseases, including cardiovascular disease and certain types of cancer [38].

Regarding photosynthetic pigments, high levels of chlorophyll b (684.7 mg/100 g DW) were identified, alongside lower levels of chlorophyll a (160.4 mg/100 g DW) and pheophytin b (74.6 mg/100 g DW). The total content of these pigments was found to be 919.7 mg/100 g DW. These pigments perform physiological functions and have been associated with antioxidant, anti-inflammatory, antitumor and anti-obesity properties, highlighting their nutritional value [39].

**Figure 2:**
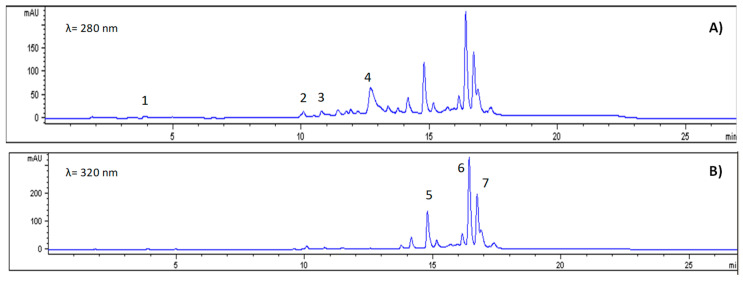
Chromatograms of phenolic compound identification at 280 nm (**A**) and 320 nm (**B**). Note: 1, Gallic acid; 2, Chlorogenic acid; 3, Caffeic acid; 4, Ferulic acid; 5, Kaempferol; 6, Quercetin glucoside; 7, Quercetin.

Phenolic compounds, a key group of secondary metabolites, have a total concentration of 5835.8 mg/100 g DW, which falls within the range reported for tropical fruits (700–5354 mg/100 g DW) [35]. The most abundant individual compounds were ferulic acid (3163.5 mg/100 g DW), quercetin glycoside (945.9 mg/100 g DW), and free quercetin (766.8 mg/100 g DW) (Figure 2). These compounds are recognised for their antioxidant activity and act as electron donors, metal chelators and modulators of endogenous antioxidant enzymes [11,40].

### 3.3. Antimicrobial Activity

Table 4 shows the antimicrobial activity of the freeze-dried ethanolic extract of ‘Star fern’ against microorganisms ATCC, such as *Escherichia coli*, *Pseudomonas aeruginosa*, *Staphylococcus aureus*, *Streptococcus mutans*, *Candida albicans* and *C. tropicalis*.

Table 5 shows the minimal inhibitory concentration of microorganisms ATCC against freeze-dried ethanolic extract of ‘Star fern’ against *E. coli*, *P. aeruginosa*, *S. aureus*, *S. mutans*, *C. albicans*, and *C. tropicalis.* In addition, Table 6 shows the minimal inhibitory concentration of multidrug-resistant bacteria against *E. faecalis*, *E. coli*, *K. pneumoniae*, *P. aeruginosa*, *S. enterica* serovar Kentucky, and *S. epidermidis*. Thus, the extract exhibited activity against two of the seven multidrug-resistant bacteria tested, *E. coli* and *P. aeruginosa*, with a MIC of 6.6 mg/mL.

Freeze-dried ethanolic extract of *G. pectinata* leaves exhibits significant inhibitory effects against *S. mutans* (0.2 mg/mL), followed by multidrug-resistant bacteria, *P. aeruginosa*, *E.coli* (6.6 mg/mL) and two non-multidrug-resistant bacteria, *P. aeruginosa* (12 mg/mL), and *S. aureus* (31.3 mg/mL). Our findings indicate that *G. pectinata* extract is a potential candidate for further investigation because the MIC for *S. mutans* is below 1 mg/mL. This finding is consistent with prior reports suggesting that plant extracts and natural products with MICs below 1 mg/mL warrant priority due to their high effectiveness against pathogens, and should be subjected to rigorous characterization [41].

Inhibiting *S. mutans* is particularly relevant as it is one of the main etiological agents of dental caries. Its pathogenicity stems from its ability to adhere to enamel, form cariogenic biofilms, produce acid from sugars and survive in highly acidic environments. These mechanisms contribute to the demineralisation of tooth enamel and ultimately to the progression of carious lesions [42]. Similarly, the activity against *S. aureus*, which is one of the most prevalent pathogens in skin and soft tissue infections and systemic processes, further highlights the value of the extract. *S. aureus* possesses multiple virulence mechanisms and can readily develop antimicrobial resistance; therefore, discovering natural compounds effective against this microorganism is a priority [43]. Inhibition was also observed against *P. aeruginosa* and *E. coli,* two bacteria that are particularly resistant in hospital environments. These bacteria cause opportunistic infections in immunocompromised patients, such as urinary tract infections, and are notable for their ability to form biofilms and express multiple resistance genes [44,45].

The antimicrobial efficacy can be partly attributed to the presence of phenolic acids such as caffeic acid (457.4 mg/100 g DW) and chlorogenic acid (104.7 mg/100 g DW). These compounds are known for their ability to disrupt bacterial cell membranes and chelate essential metals. These mechanisms inhibit microbial growth and can enhance the action of other bioactive compounds. Other phenolic acids, such as gallic acid and cinnamic acid, have also demonstrated antimicrobial properties, contributing to a synergistic effect in the plant extract [14].

Among the flavonoids, quercetin (766.8 mg/100 g DW) and its glucoside (945.9 mg/100 g DW) have been documented as being effective against *S. aureus* by inhibiting essential enzymes, altering membrane integrity, and suppressing biofilm formation [15]. Another relevant compound is malic acid (56.6 mg/100 g DW), which contributes to the extract’s acidic pH, creating an environment that is unfavourable for microbial growth [37].

### 3.4. Antioxidant Activity

Table 7 presents the results of the antioxidant activity tests, conducted using the ABTS and DPPH methods. The antioxidant activity of *G. pectinata* leaves was moderate, as indicated by values of 5.5 mmol TE/100 g DW in the DPPH assay and 6.5 mmol TE/100 g DW in the ABTS assay. This indicates the presence of compounds that can neutralise free radicals, albeit at concentrations or in structures that are possibly less reactive than those of other highly antioxidant species. The particular characteristics of the assays explain the difference between the two methods. The DPPH assay measures the hydrogen-donating capacity of antioxidants that contain hydroxyl groups. In contrast, the ABTS assay is more versatile, enabling the detection of both hydrophilic and lipophilic antioxidants and demonstrating greater sensitivity to a broader spectrum of bioactive compounds [18].

While the antioxidant values were not exceptionally high, they reflect a plant matrix with functional potential, supported by the presence of various secondary metabolites with recognised redox activity. Among the identified compounds, ferulic acid was the most abundant phenol (3163.5 mg/100 g DW), and it is considered a powerful antioxidant due to its ability to stabilise free radicals through hydrogen donation. In turn, despite being present in smaller quantities, gallic acid (5.5 mg/100 g DW) also contributes significantly due to its high density of hydroxyl groups, which give it remarkable redox efficiency. Similarly, flavonoids such as quercetin (766.8 mg/100 g DW), its glycoside (945.9 mg/100 g DW), and kaempferol (391.9 mg/100 g DW) exhibit strong antioxidant properties, neutralising free radicals directly and modulating endogenous antioxidant enzymes. Previous studies have shown that flavonoids such as rutin and kaempferol play important roles in protecting against cellular oxidative damage [46].

Among the pigments with antioxidant activity, β-carotene (266.6 mg/100 g DW) is notable, as it is a lipophilic carotenoid that protects membrane lipids from peroxidation and acts as a precursor of vitamin A, which is essential for immune processes and visual health. Additionally, the high levels of chlorophylls (919.7 mg/100 g DW in total) reinforce this capacity as chlorophyll is involved in deactivating singlet oxygen.

Various ferns have also been found to contain flavonoids, polyphenols, carotenoids, terpenoids, steroids and polyunsaturated fatty acids. These compounds have been linked to antioxidant and antimicrobial activities [8]. In the case of *G. pectinata* specifically, the presence of these compounds in varying concentrations suggests a synergistic effect that explains the recorded antioxidant activity, positioning this species as a promising candidate for preventive health applications.

### 3.5. Antitumor Activity

The antiproliferative activity of the freeze-dried ethanolic extract of *G. pectinata* leaves was assessed using the MTT assay across a panel of tumour cell lines—HeLa, HCT116, THJ29T, and HepG2—as well as non-tumorigenic NIH3T3 fibroblasts (Table 8).

The freeze-dried ethanolic extract of *G. pectinata* leaves demonstrated dose-dependent cytotoxicity across all tested tumour cell lines, with IC_50_ values ranging from 0.98 to 1.98 mg/mL. Among the cell lines, HepG2 (hepatocellular carcinoma) cells exhibited the highest sensitivity (IC_50_ = 0.98 mg/mL), followed by HeLa (cervical cancer, IC_50_ = 1.17 mg/mL), THJ29T (thyroid carcinoma, IC_50_ = 1.70 mg/mL), and HCT116 (colorectal cancer, IC_50_ = 1.98 mg/mL), indicating variable responsiveness depending on tumor type. These results indicate moderate antiproliferative potency for a crude extract and suggest that the bioactive constituents of *G. pectinata* may act through mechanisms differentially effective across tumour origins. In comparison, the IC_50_ values reported for the standard chemotherapeutic agent cisplatin were lower [47].

To our knowledge, these data provide initial evidence of in vitro antitumor activity for a freeze-dried ethanolic extract of *G. pectinata* leaves, marking a significant contribution to the largely uncharted pharmacological profile of this fern. The extract’s ability to reduce cancer cell viability in vitro suggests that this species produces compounds with biological activity relevant to tumour suppression. Although reports on Gleicheniaceae are scarce, related ferns, such as *Pteridaceae* and *Polypodiaceae*, exhibited antitumor-relevant effects, including apoptosis induction, anti-inflammatory modulation, and chemopreventive activity [48,49,50]. Within the family itself, *Dicranopteris linearis* has exhibited notable cytotoxicity against HL-60 and MDA-MB-231 cancer cell lines, and both this species and *Gleichenia quadripartita* have yielded bioactive phenolic and diterpenoid metabolites, including diterpenoid glycosides characteristic of *Gleicheniaceae* fern constituents [51,52,53]. Supporting this chemical profile, recent reviews highlight fern secondary metabolites as promising sources of pharmacologically active compounds [54].

The therapeutic index (TI), calculated relative to non-tumour NIH3T3 fibroblasts, further highlighted these differences. The highest TI was observed in HepG2 cells (TI = 1.5), suggesting comparatively better selectivity. In contrast, the TI for HCT116 was notably low (0.8), reflecting reduced selectivity and potential off-target toxicity. Overall, the extract displayed moderate cytotoxic potency for a crude preparation, though the narrow TI values (≤1.5) indicate limited tumour-specific selectivity that warrants fractionation before claiming therapeutic promise.

The observed differences in sensitivity across cancer cell lines imply that the extract’s components may act through mechanisms more effective in specific tumour types, notably hepatocellular and cervical carcinomas. However, the lack of marked selectivity over non-tumour fibroblasts raises concerns about potential off-target effects. This limitation is standard in initial screenings of crude extracts, which often contain complex mixtures of compounds with diverse and sometimes opposing bioactivities [55].

The phytochemical composition of the freeze-dried ethanolic extract of *G. pectinata* leaves provides a biologically plausible basis for the antitumor effects observed in vitro. Although the extract displayed only moderate antioxidant activity, its high levels of phenolic acids (notably ferulic and caffeic), flavonoids (such as quercetin and kaempferol derivatives), and carotenoids are noteworthy, as these compounds have been associated with mechanisms including apoptosis induction, modulation of oncogenic signalling pathways, and interference with tumour microenvironment dynamics. However, despite this rich profile of phenolic and organic acids, its moderate cytotoxic potency underscores that total phytochemical abundance alone does not guarantee strong biological activity. Rather, the antitumor efficacy of compounds like caffeic and ferulic acid is known to depend on their structural specificity, cellular uptake, and molecular targets [56,57]. This highlights the importance of identifying which individual constituents or synergistic interactions are truly responsible for the effects observed.

### 3.6. Anti-Inflammatory Activity

**Figure 3:**
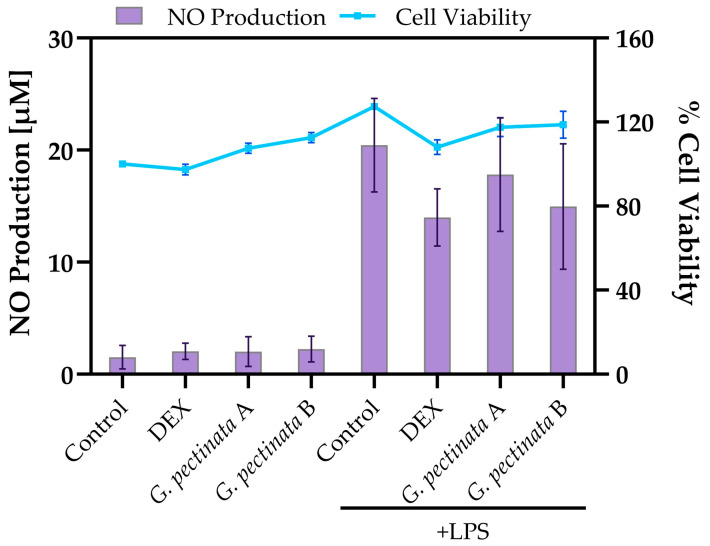
Effects of freeze-dried ethanolic extract of *G. pectinata* leaves (A, 0.05 mg/mL; B, 0.1 mg/mL) on RAW264.7 macrophages. Note: Nitric oxide production (**left** *y*-axis, bars) and cell viability (**right** *y*-axis, line) under the same conditions. Data are expressed as mean ± SD (n = 3). No statistically significant differences were observed between *G. pectinata* leaves + LPS treatments and the LPS-stimulated control.

RAW 264.7 macrophages were treated with two concentrations (A and B) of the freeze-dried ethanolic extract of *G. pectinata* leaves to assess its effects under both LPS-stimulated and unstimulated conditions. Upon LPS stimulation, treatment with freeze-dried ethanolic extract of *G. pectinata* leaves reduced nitric oxide (NO) production in a concentration-dependent manner (17.8 µM and 15.0 µM for concentrations A and B, respectively; Figure 3). This anti-inflammatory effect was less pronounced than the positive control, dexamethasone (DEX, 14.0 µM). As expected, the LPS control group exhibited the highest NO release (20.4 µM). In unstimulated cells, NO levels remained basal (2.0–2.2 µM) and were comparable to the medium-only control (1.5 µM).

**Figure 4:**
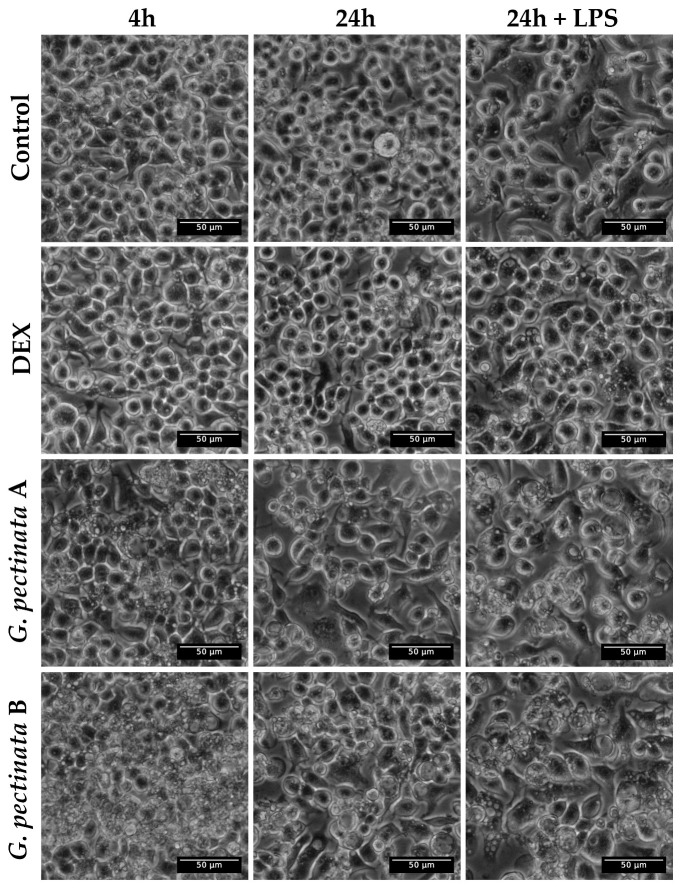
Morphological changes at 4 h and 24 h with or without LPS of freeze-dried ethanolic extract of *G. pectinata* leaves (A, 0.05 mg/mL; B, 0.1 mg/mL) on RAW264.7 macrophages.

Notably, the extract induced significant morphological alterations, with vacuolization observed as early as 4 h post-treatment at the lowest concentration (A) and persisting for 24 h in both extract-treated groups, irrespective of LPS stimulation (Figure 4). In contrast, cells in the medium-only control and dexamethasone (DEX)-treated groups maintained normal morphology at both time points. In contrast, vacuolization in the LPS control was only observed after 24 h. Importantly, these morphological alterations were observed even though cell viability remained consistently above 95% in all groups. Extract-treated cells reached marginally higher values (117.5% at concentration A and 118.7% at concentration B) relative to the medium-only control, which was set as 100% viability.

Among the bioactive constituents identified, ferulic acid emerged as the most abundant phenolic compound in *G. pectinata* leaf extract. It may underlie the moderate suppression of nitric oxide (NO) production observed in LPS-stimulated RAW 264.7 macrophages. This observation is consistent with previous reports that ferulic acid mediates anti-inflammatory responses through autophagy induction and inhibition of the NLRP3 inflammasome, leading to the downregulation of pro-inflammatory cytokines (IL-1β, IL-6, TNF-α) and mediators (iNOS, COX-2) [58,59]. Another key phenolic constituent identified, chlorogenic acid, exerts complementary effects by blocking NF-κB nuclear translocation and activating the Nrf2 pathway, thereby reducing the expression of pro-inflammatory cytokines (TNF-α, IL-1β, IL-6) and enzymes (iNOS, COX-2), while promoting the release of anti-inflammatory interleukins such as IL-4, IL-10, and IL-13 [60,61].

Other phenolic constituents, including gallic acid, kaempferol, and quercetin, further contribute to the anti-inflammatory profile of the extract. Notably, quercetin has been reported to induce morphological alterations in macrophages concurrent with NO suppression, a phenomenon that aligns with the cellular disruptions observed in the present study [62,63]. Alongside phenolic compounds, carotenoids such as β-carotene and lutein were also detected in the extracts, which are known to regulate inflammatory responses by modulating cytokine expression and interfering with critical signaling pathways, including NF-κB [64,65].

Interestingly, related species within the *Gleicheniaceae* family, such as *Gleichenia truncata*, have also demonstrated anti-inflammatory and, additionally, anti-malarial activities. These effects have been linked to inhibition of glycogen synthase kinase-3β (GSK3β) and suppression of key cytokines such as TNF-α and IFN-γ during *Burkholderia pseudomallei* infection [9], suggesting that conserved bioactive mechanisms may exist within this plant family.

## 4. Conclusions

*Gleichenella pectinata*, known as ‘Star fern’, is a species traditionally used by Amazonian indigenous communities to treat various diseases; however, scientific information on its composition and bioactivity is limited. In this study, the leaves showed low titratable acidity, correlated with a high concentration of organic acids, especially malic acid. In addition, high levels of potassium, calcium, and iron were detected. The screening showed the presence of acetogenins, flavonoids, and phenols. Among the pigments, β-carotene was the predominant carotenoid, and chlorophyll b had the highest concentration. In terms of phenolic compounds, ferulic acid and quercetin glucoside stood out for their high values. Antioxidant activity (ABTS and DPPH) showed similar results with significant activities. The freeze-dried ethanolic extracts were more effective against *Pseudomonas aeruginosa* ATCC and multi-resistant bacteria such as *Escherichia coli and P. aeruginosa.* Finally, moderate antitumor potential was observed in liver and cervical carcinoma cells, attributable to its profile of phenolic acids, flavonoids, acetogenins and carotenoids, positioning *G. pectinata* as a promising candidate for future research into natural therapies.

## Figures and Tables

**Table 1:** Average values of the chemical characteristics of ‘Star fern’.

Parameters	Leaves		
pH	4.6	±	0.1
Soluble solids (°Brix)	1.0	±	0.0
Titratable acidity (%)	2.4	±	0.3
Humidity (%)	63.9	±	0.5
Ash (%)	4.5	±	0.8
Mineral profile (mg/100 g DW)			
Ca	245.4	±	6.1
Fe	95.2	±	1.2
K	877.5	±	5.0
Mg	47.4	±	1.0
Na	0.2	±	0.0

**Table 2:** Phytochemical screening of ‘Star fern’.

Metabolites	Leaves
Acetogenins	+
Alkaloids	−
Anthraquinones	−
Flavonoids	+
Phenols	+
Saponins	−
Steroids	+
Tannins	+
Terpenoids	+

Note: −, Negative test result; +, Positive test result.

**Table 3:** Average values of bioactive compounds of ‘Star fern’.

Parameters	Leaves		
Vitamin C (mg/100 g DW)	16.6	±	1.1
Organic acid (mg/100 g DW)			
Citric acid	162.1	±	3.5
Malic acid	56,559.7	±	43.9
Tartaric acid	14.4	±	0.7
Total organic acid	56,736.2	±	41.1
Carotenoids (mg/100 g DW)			
Lutein	112.9	±	8.6
Violaxanthin	15.4	±	3.3
α-Carotenoid	0.8	±	0.1
β-Carotenoid	266.6	±	5.0
Carotenoid total	336.2	±	6.6
Chlorophylls and their derivatives (mg/100 g DW)			
Chlorophyll a	160.4	±	7.0
Chlorophyll b	684.7	±	20.3
Pheophytin b	74.6	±	7.2
Total chlorophyll	919.7	±	7.2
Phenolic compounds (mg/100 g DW)			
Gallic acid	5.5	±	0.4
Chlorogenic acid	104.7	±	9.7
Caffeic acid	457.4	±	21.1
Ferulic acid	3163.5	±	16.9
Kaempferol	391.9	±	1.9
Quercetin glucoside	945.9	±	15.9
Quercetin	766.8	±	4.1
Total phenolics	5835.8	±	14.5

**Table 4:** Average values of the antimicrobial activity of microorganisms ATCC against freeze-dried ethanolic extract of ‘Star fern’.

Microorganisms	Zona of Inhibition (mm)		
*Escherichia coli* ATCC 8739	-		
*Pseudomonas aeruginosa* ATCC 9027	10.0	±	0.0
*Streptococcus aureus* ATCC 6538P	19.8	±	2.9
*Streptococcus mutans* ATCC 25175	23.5	±	0.7
*Candida albicans* ATCC 1031	-		
*Candida tropicalis* ATCC 13803	-		

Note: - indicates no activity at the tested concentrations.

**Table 5:** Minimal inhibitory concentration of microorganisms ATCC against freeze-dried ethanolic extract of ‘Star fern’.

Microorganisms	Minimal Inhibitory Concentration (mg/mL)
*Escherichia coli* ATCC 8739	-
*Pseudomonas aeruginosa* ATCC 9027	12.0
*Streptococcus aureus* ATCC 6538P	31.3
*Streptococcus mutans* ATCC 25175	0.2
*Candida albicans* ATCC 1031	-
*Candida tropicalis* ATCC 13803	-

Note: - indicates no activity at the tested concentrations.

**Table 6:** Minimal inhibitory concentration of multidrug-resistant bacteria against freeze-dried ethanolic extract of ‘Star fern’.

Microorganisms *	Minimal Inhibitory Concentration (mg/mL)
*Enterococcus faecalis*	-
*Escherichia coli*	6.6
*Klebsiella pneumoniae*	-
*Pseudomona aeruginosa*	6.6
*Salmonella enterica* serovar Kentucky	-
*Staphylococcus epidermidis*	-

Note: - indicates no activity at the tested concentrations; * multidrug-resistant bacteria.

**Table 7:** Average values of antioxidant activity of ‘Star fern’ in leaves.

Parameters	Antioxidant Activity (mmol TE/100 g DW)		
DPPH	5.5	±	0.1
ABTS	6.5	±	0.3

**Table 8:** Half maximal inhibitory concentration values (IC_50_) (mg/mL) of star fern against tumour and non-tumour cell lines at 72 h and therapeutic index (TI) values. Values are expressed as mean ± standard deviation, n = 4.

	HeLa	HCT116	THJ29T	HepG2	NIH3T3
IC_50_	1.17 ± 0.20	1.98 ± 0.21	1.70 ± 0.26	0.98 ± 0.33	1.50 ± 0.80
TI ^a^	1.3	0.8	0.9	1.5	--

^a^ IC_50_ = IC_50_ (NIH3T3)/IC_50_ (tumor cell).

## Data Availability

The original contributions presented in this study are included in the article. Further inquiries can be directed to the corresponding author.
